# Therapeutic Handbook of the U. S. Pharmacopœia

**Published:** 1883-08

**Authors:** 


**Therapeutic Handbook of the U. S. Pharmacopœia,** being a condensed statement of the physiological and toxic action, medicinal value, methods of administration and doses of the drugs, and preparation in the latest edition of the United States Pharmacopœia (Apothecaries' and Metric systems), with some remarks on unofficinal preparations. By ROBERT T. EDES, B. A., M. D. New York: Wm. Wood & Co. 1883.

As the title indicates, the book is an abbreviated Dispensatory. The author states that he has endeavored to supply, briefly and concisely, the information necessary to make it a convenient book for the practitioner, and in this he has succeeded. It cannot be expected that in a book of 400 pages he has been able to condense all the therapeutic knowledge contained in the immense tomes devoted to pharmacology, but he has made the condensation with good judgment, and presents to the profession a useful book. Indeed, any book is valuable which will aid in familiarizing the medical profession with the contents of the Pharmacopœia, so that they may recognize how well all possible therapeutic demands are therein provided for, and how unnecessary it is that they should undergo the humiliation of seeing their patients, whose wants they are supposed to know, prescribed for by distant druggists, who manufacture complicated preparations with high-sounding names. Most of these are very distinctly inferior to the simpler material of the Pharmacopœia, open to all without concealment, advertisement or charlatanry of any kind.

